# A Collyrium for Ophthalmia Neonatorum

**Published:** 1898-02

**Authors:** 


**A Collyrium for Ophthalmia Neonatorum.**—The *Gazette Hebdomadaire de Medicine et de Chirurgie* gives the following formula:

| | | |
|---|---|---|
| ℞ | Hydrastine sulphate | 1 part. |
| | Boric acid | 1 part. |
| | Sodium biborate | 1 part. |
| | Tincture of opium | 1 part. |
| | Distilled water | 100 parts. |

M. A few drops to be instilled into the eyes every hour. In the intervals the eyes may be irrigated with warm boiled water and a little vaseline applied to the edges of the lids.—*New York Medical Journal.*

